# Continuous Glucose and Heart Rate Monitoring in Young People with Type 1 Diabetes: An Exploratory Study about Perspectives in Nocturnal Hypoglycemia Detection

**DOI:** 10.3390/metabo11010005

**Published:** 2020-12-24

**Authors:** Valeria Calcaterra, Pietro Bosoni, Lucia Sacchi, Gian Vincenzo Zuccotti, Savina Mannarino, Riccardo Bellazzi, Cristiana Larizza

**Affiliations:** 1 Pediatric and Adolescent Unit, Department of Internal Medicine, University of Pavia, 27100 Pavia, Italy; valeria.calcaterra@unipv.it; 2 Pediatric Unit, Department of Pediatrics, Children’s Hospital “V. Buzzi”, 20157 Milano, Italy; gianvincenzo.zuccotti@unimi.it; 3 Department of Electrical, Computer and Biomedical Engineering, University of Pavia, 27100 Pavia, Italy; pietro.bosoni02@universitadipavia.it (P.B.); lucia.sacchi@unipv.it (L.S.); riccardo.bellazzi@unipv.it (R.B.); 4 Department of Biomedical and Clinical Sciences “L. Sacco”, University of Milan, 20157 Milano, Italy; 5 Pediatric Cardiology Unit, Department of Pediatrics, Children’s Hospital “V. Buzzi”, 20157 Milano, Italy; savina.mannarino@asst-fbf-sacco.it

**Keywords:** nocturnal hypoglycemia, type 1 diabetes, heart rate variability, flash glucose monitoring

## Abstract

A combination of information from blood glucose (BG) and heart rate (HR) measurements has been proposed to investigate the HR changes related to nocturnal hypoglycemia (NH) episodes in pediatric subjects with type 1 diabetes (T1D), examining whether they could improve hypoglycemia prediction. We enrolled seventeen children and adolescents with T1D, monitored on average for 194 days. BG was detected by flash glucose monitoring devices, and HR was measured by wrist-worn fitness trackers. For each subject, we compared HR values recorded in the hour before NH episodes (before-hypoglycemia) with HR values recorded during sleep intervals without hypoglycemia (no-hypoglycemia). Furthermore, we investigated the behavior after the end of NH. Nine participants (53%) experienced at least three NH. Among these nine subjects, six (67%) showed a statistically significant difference between the before-hypoglycemia HR distribution and the no-hypoglycemia HR distribution. In all these six cases, the before-hypoglycemia HR median value was higher than the no-hypoglycemia HR median value. In almost all cases, HR values after the end of hypoglycemia remained higher compared to no-hypoglycemia sleep intervals. This exploratory study support that HR modifications occur during NH in T1D subjects. The identification of specific HR patterns can be helpful to improve NH detection and prevent fatal events.

## 1. Introduction

Hypoglycemia is a common and serious side effect of insulin therapy in subjects with type 1 diabetes (T1D) [1,2,3,4]. Early detection of hypoglycemia may improve treatment and avoidance of severe complications, including dead-in-bed syndrome, which is an unexpected death during sleep [5,6,7,8,9]. 

Dead-in-bed syndrome occurs in about 5% of deaths in childhood-onset diabetes [10]; hypoglycemia, ECG abnormalities, and autonomic dysfunction have been suggested to be involved in the pathogenic mechanisms. Cardioacceleration and corrected QT (QTc) prolongation have been reported during hypoglycemia as a dominant sympathoadrenal response. During episodes of nocturnal hypoglycemia (NH), different phases of heart rate (HR) variability are reported, supporting that the initial sympathetic activity to hypoglycemia can be followed by a parasympathetic response [11]. A risk of arrhythmic death can occur in patients with cardiovascular risk and autonomic failure due to a blunted nocturnal sympathoadrenal response and relative parasympathetic predominance. However, measurable changes in HR variability during hypoglycemia are present in subjects both with and without cardiovascular autonomic neuropathy (CAN) [12]. Nevertheless, it remains not fully elucidated how CAN influences these measurable changes.

Recently, a combination of information from continuous glucose monitor (CGM) and HR measurements have been proposed to predict hypoglycemia in both bedbound and active T1D subjects [13,14]. This study aims to investigate the HR changes during nocturnal hypoglycemic episodes in children and adolescents with T1D, examining whether such signals could improve the detection and prevention of NH. For this purpose, blood glucose (BG) levels were monitored by a flash glucose monitoring (FGM) device, and HR was recorded by a wrist-worn personal fitness tracker (PFT), both connected to the Advanced Intelligent Distant-Glucose Monitoring (AID-GM) web-based platform [15]. Results from this study will provide important knowledge for the development of a potential NH alert.

## 2. Results

Baseline characteristics of the participants at the time of recruitment are presented in Table 1. Subjects were monitored on average for 194 days (± standard deviation of 83 days). Overall, 68,560 h of BG monitoring and 15,392 h of simultaneous BG and HR monitoring were recorded. A total of 773 sleep intervals were detected, including 170 h of BG recordings during sleep in the hypoglycemic range, i.e., where BG < 70 mg/dL (BG < 3.9 mmol/L) [16]. For each subject, the median duration of hypoglycemic episodes during sleep was 57 min, with an interquartile range of 43 min.

Nine of the seventeen participants experienced at least three episodes of hypoglycemia during sleep. A total of 516 valid sleep intervals remained after the preprocessing step, split into 68 intervals (13%) with the occurrence of hypoglycemic episodes and 448 intervals (87%) without hypoglycemic episodes. 

**Figure 1:**
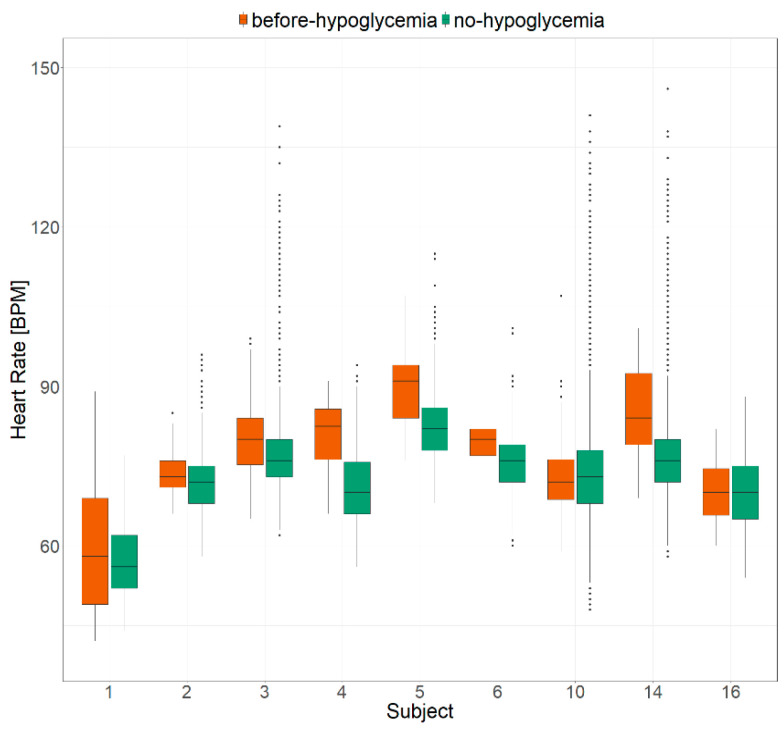
Comparison between the heart rate values collected in the hour before a hypoglycemic episode (in red) and in sleep intervals without hypoglycemic episodes (in green).

For each of the nine selected subjects, the distribution of HR values in the before-hypoglycemia dataset was compared to the distribution of HR values in the no-hypoglycemia dataset (Figure 1). All subjects but three (Subjects 1, 10, and 16) showed a statistically significant difference between the before-hypoglycemia HR distribution and the no-hypoglycemia HR distribution, with a *p*-value < 0.01. Interestingly, in all these six cases (67%), the HR median value of the before-hypoglycemia dataset was higher than the HR median value of the no-hypoglycemia dataset. On average, the before-hypoglycemia HR values were higher than the HR median value of the respective no-hypoglycemia dataset in 75% of cases (± standard deviation of 14%). Subject 1 also showed a higher HR median value before hypoglycemic episodes than in sleep intervals without hypoglycemia, but the difference is not statistically significant, while for Subjects 10 and 16, the HR median values were comparable. 

**Figure 2:**
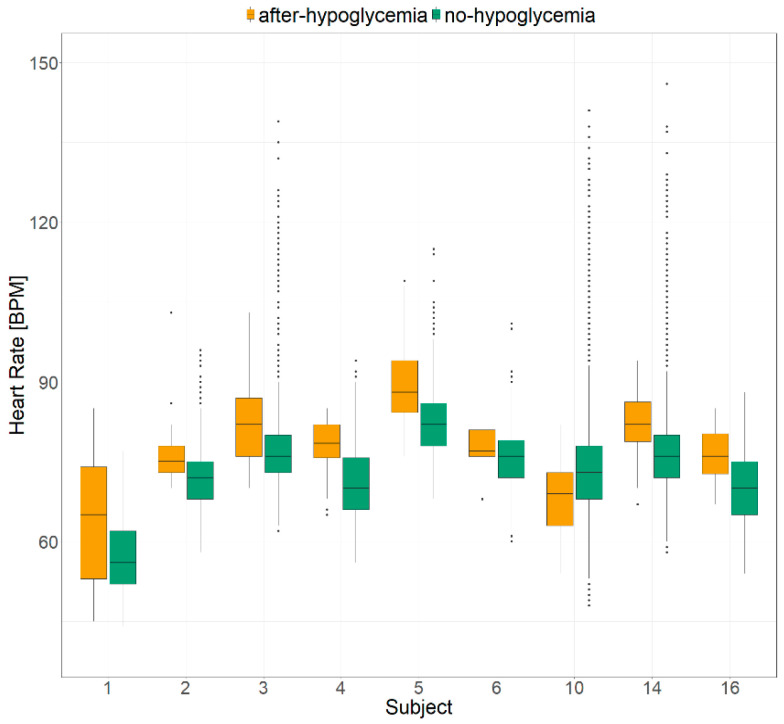
Comparison between the heart rate values collected in the hour after a hypoglycemic episode (in orange) and in sleep intervals without hypoglycemic episode (in green).

Figure 2 displays a comparison between the HR distribution in after-hypoglycemia and no-hypoglycemia datasets for each subject. The HR median values in the hour after the end of hypoglycemia were higher than in sleep intervals without hypoglycemia for all cases but one (Subject 10), with a statistically significant difference in six cases (67%).

## 3. Discussion

T1D is a common chronic disease in children and adolescents, caused by auto-immune destruction of beta-pancreatic cells and characterized by chronic hyperglycemia [17,18]. A T1D self-management requires frequent BG levels checking, carbohydrate (CHO) counting, evaluation of the affective response to exercises, and insulin self-injecting [17,18,19].

A reduction in the incidence and progression of diabetes-related complications is associated with tight glycemic control [20,21]; nevertheless, the improvement of glycemic control increased the risk of iatrogenic hypoglycemia [1,2,3,4]. The main part of excess mortality in T1D is related to long-term complications; however, an excess death rate has also been reported in subjects with short disease duration and without long-term adverse effects [22,23,24]. Part of this mortality has been attributed to an unexpected death during sleep, related to NH [25]. Although the pathogenic mechanism of the dead-in-bed syndrome is not fully understood, plausible theories support the role of hypoglycemic episodes, cardiac arrhythmias, cardiac autonomic failure, or a combination of these [6,26,27,28,29,30,31].

In experimental studies, the morphological changes in electrocardiographic repolarization, the QTc prolongation, and cardiac rate/rhythm disturbances have been described during hypoglycemia in T1D [5,8,32].

Abnormal cardiac repolarization during hypoglycemia appears to be mediated by both the direct effect of sympathoadrenal stimulation and catecholamine- and insulin-induced hypokalemia on cardiac ion channels [27]. Hypoglycemia is recognized as a potential proarrhythmic event [31], but direct evidence linking electrocardiographic changes, and the dead-in-bed syndrome is still missing [30]. Multiple factors such as overt or undetected autonomic neuropathy, genetic contribution, or abnormally intensive sympathoadrenal response are likely to contribute to the phenomenon [31]. However, the disturbance of cardiac rhythm may represent a crucial point in the study of NH for preventing fatal outcomes.

In this study, we confirmed that symptomatic or asymptomatic hypoglycemia continues to frequently occur in young people with T1D during sleep. Considering subjects with at least three episodes of NH, in 67% of cases, there was a statistically significant difference between the distribution of the HR values collected in the hour before a hypoglycemic episode and the distribution of the HR values collected in sleep intervals without hypoglycemia. In addition, for all the subjects that showed a significant difference between the two HR distributions, the HR median value of the before-hypoglycemia dataset was higher than the HR median value of the no-hypoglycemia dataset. In addition, after the end of the hypoglycemic episode, in all cases but one, the HR values remained higher compared to those collected in the no-hypoglycemia sleep intervals. 

The presence of higher HR values in the before-hypoglycemia dataset supports the sympathetic response to hypoglycemia [33], while a persistent increase in HR could be implicated in hypoglycemia-induced cardiac arrhythmias. Increased HR followed by an incorrect adjustment of repolarization, with inhomogeneous prolongation of the action potential duration, can lead to the dispersion of ventricle repolarization and fatal arrhythmias [33]; the additional role of renin-angiotensin system activity in the magnitude of the adrenaline response to hypoglycemia could also be considered in subjects with T1D [34]. A subsequent increase of the parasympathetic activity to defend the organism cannot be excluded, leading to a risk for fatal heart rhythm problems in subjects with autonomic failure.

Our results suggest that HR may be an interesting parameter to discern the episodes of hypoglycemia. Simultaneous monitoring of various physiological parameters could offer unexplored opportunities to build algorithms for early detection of hypoglycemic events, as described by Bertachi et al. in a cohort of adult subjects [35]. The introduction of a hypoglycemia prevention algorithm can improve glycemic outcome measures [36], and the optimization of glycemic control may improve cardiac sympathetic and parasympathetic activities, reducing the risk for a fatal alteration of rhythm [37]. Finally, data confirm that the identification of physiological responses that are non-invasively measurable with a wearable sensor system can have a potential role for disease management in young people with T1D [38,39]. 

We acknowledge several limitations of our study. The number of participants is relatively small, not enough for the development of a predictive model; in the future, increased sample sizes are recommended. We selected a group of T1D subjects who were free of complications since older participants and those with early complications could have more evident HR alterations. In addition, we have not data about morphological changes in electrocardiographic repolarization and/or the QTc prolongation, which could be useful to study the pathogenetic mechanisms of cardiac arrhythmias. Finally, the FGM systems may be less accurate than direct glucose measurements, although the Abbott FreeStyle Libre sensor has been validated in children and adolescents with T1D [40]. 

In conclusion, this exploratory study supports that HR modifications occur during NH in T1D subjects. The identification of specific HR patterns could be helpful to improve the early detection of hypoglycemic episodes and prevent fatal events. The creation of a NH prediction algorithm may represent a challenge for the management of diabetes in children and adolescents.

## 4. Materials and Methods

### 4.1. Subjects

A group of seventeen children and adolescents with T1D under multiple daily injections, using a rapid-acting insulin analog such as prandial insulin and a basal insulin analog, was recruited from the Pediatric Diabetology Unit at Fondazione IRCCS Policlinico San Matteo Hospital in Pavia, Italy, between August and December 2018. Subjects with retinopathy, nephropathy, established macrovascular disease, and those on drugs likely to affect cardiac function or rhythm were excluded.

Anthropometric data were recorded at the enrollment. A FGM sensor (FreeStyle Libre^®^, Abbott Diabetes Care, Alameda, CA, USA) [41] and a PFT device (Fitbit Charge HR^®^, San Francisco, CA, USA) [42] were delivered to the study participants, who were asked to periodically upload their monitoring data through the AID-GM application with the help of their caregivers. Participants were instructed to apply the FGM sensor at the back of the upper arm and change it every fourteen days. The PFT is a wrist-worn device resembling a watch, which uses photoplethysmography (PPG) to detect periodic changes in blood flow beneath the sensor, thereby measuring HR.

The study was performed in accordance with the Declaration of Helsinki, and the protocol was approved by the Institutional Review Board of the hospital. Children’s caregivers (or subjects aged ≥ 18 years) provided written consent for inclusion after they were given information about the nature of the study.

### 4.2. Anthropometric and Clinical Assessment

Physical examination of subjects included anthropometric measurements of weight and height, Body Mass Index (BMI) calculation, and assessment of the pubertal stage.

Weight was measured with participants in light clothing without shoes, standing upright in the center of the scale platform facing the recorder, hands at sides, and looking straight ahead. For standing height, subjects were instructed to stand as tall as possible in an upright position without shoes, with heels together and toes apart, hands at sides, aligning the head in the Frankfort horizontal plane, and taking a deep breath. Measurements were performed using a Harpenden stadiometer with a fixed vertical backboard and an adjustable headpiece.

BMI was calculated by dividing the subjects’ weight in kilograms by the square of the height in meters. The Marshall and Tanner scale was used to evaluate the pubertal staging, with the pre-pubertal characteristics corresponding to Tanner Stage 1 [43,44].

### 4.3. Advanced Intelligent Distant—Glucose Monitoring (AID-GM)

AID-GM is a web application developed at the Department of Electrical, Computer and Biomedical Engineering of the University of Pavia, Italy, in collaboration with the Pediatric Diabetology outpatient service of the Fondazione IRCCS Policlinico San Matteo Hospital in Pavia [15]. The AID-GM platform has been designed for the integration of heterogeneous data coming from FGM sensors, PFTs, and vocal messages provided by subjects reporting daily diary information, useful for monitoring both lifestyle and metabolic control of diabetic subjects. A joint analysis of BG, HR, and activity data, in fact, can provide information about the glycemic profile and help to contextualize the occurrence of relevant episodes during daily routines, workouts, or sleep.

The AID-GM application offers a wide range of visualization and innovative analytical tools. In particular, it supports advanced temporal data analysis functionalities for the extraction of qualitative patterns from time-series data. This is possible thanks to the integration with the Java Time Series Abstractor (JTSA), a framework for processing temporal data and extracting knowledge-based patterns [45]. JTSA modular structure allows combining different algorithms to extract multivariate and complex patterns that can be personalized using subject-specific parameters. Moreover, the temporal analysis can be focused on specific time frames, e.g., days of the week or moments of the day. AID-GM currently supports ten types of patterns representing well-known clinical phenomena, which could identify potentially risky situations. For example, it is possible to automatically extract the NH pattern, i.e., BG measures below the subject-specific hypoglycemia threshold during a sleep interval.

The application is designed to analyze both an individual subject and a group of subjects. This can be valuable, especially considering the huge amount of longitudinal data that has become available with the use of monitoring devices, which will not allow easy manual identification of critical situations. Statistics on a group of patients can help the physician to quickly identify subjects who need closer control and possible therapy adjustments.

### 4.4. Statistical Analysis

Exploiting the synchronization between PFT and FGM, the AID-GM platform allowed both identifying the start and endpoints of sleep intervals and detecting those intervals characterized by the occurrence of hypoglycemic patterns, i.e., where BG < 70 mg/dL (BG < 3.9 mmol/L) [16]. 

In the preprocessing step, a filter was applied to exclude all the sleep intervals that were preceded by hypoglycemic episodes in the previous two hours since these episodes could alter the succeeding BG and HR measurements. Therefore, only the HR measures recorded in the remaining sleep intervals were considered in the study. In addition, subjects with less than three overall hypoglycemic patterns during sleep were excluded.

For each selected subject three HR datasets were created, called before-hypoglycemia, after-hypoglycemia, and no-hypoglycemia. The first two datasets included HR measurements collected during sleep intervals with hypoglycemic episodes, while the last one related to sleep intervals without hypoglycemic episodes. Regarding sleep intervals with hypoglycemia, the before-hypoglycemia dataset was limited to HR measurements recorded in the hour before the hypoglycemic episode, whereas the after-hypoglycemia dataset included HR measurements recorded in the hour after the hypoglycemic episode. For nights without hypoglycemia, the analysis was performed on HR measurements collected during the whole sleep period, excluding the first hour after falling asleep and the last hour before waking up. Finally, the HR measurements from each dataset were aggregated into separate five-minute time intervals to capture the average values.

For each subject, the non-parametric Mann-Whitney U test was performed to compare whether the HR distribution was the same for before-hypoglycemia and no-hypoglycemia datasets or there was a difference between them, setting the significance threshold to 0.05. In addition, by comparing the HR distribution between after-hypoglycemia and no-hypoglycemia datasets, we investigated what happened after the end of hypoglycemic episodes. All the analyses were implemented with the R for statistical computing, version 3.5.1. Mann–Whitney U test was implemented through the function “wilcox.test” available in the R package called “stats” [46].

## Figures and Tables

**Table 1:** Study participant’s characteristics at baseline. Summary statistics are presented as frequency (percentage) and mean ± standard deviation. HbA1c: glycated hemoglobin; BMI: Body Mass Index.

Characteristic	Summary Statistics
Sex	Female: 9 (52.94%)
	Male: 8 (47.06%)
Pubertal stage	Pre-pubertal: 5 (29.41%)
	Peri-pubertal: 7 (41.18%)
	Post-pubertal: 5 (29.41%)
Age (years)	12.30 ± 4.33
Diabetes duration (years)	5.11 ± 4.18
Insulin dose (IU/Kg/die)	0.84 ± 0.24
HbA1c (%)	8.21 ± 1.29
HbA1c (mmol/mol)	66.12 ± 14.16
BMI (Kg/m^2^)	19.88 ± 5.06
Systolic arterial pressure (mmHg)	105.53 ± 11.01
Diastolic arterial pressure (mmHg)	67.65 ± 6.40

## Data Availability

The data that support the findings of this study are available on request from the corresponding author, C.L.
